# Some Properties of Electron Beam-Irradiated Sheep Wool Linked to Cr(III) Sorption

**DOI:** 10.3390/molecules24234401

**Published:** 2019-12-02

**Authors:** Jana Braniša, Angela Kleinová, Klaudia Jomová, Radka Malá, Volodymyr Morgunov, Mária Porubská

**Affiliations:** 1 Constantine the Philosopher University in Nitra, Faculty of Natural Sciences, Department of Chemistry, Tr. A. Hlinku 1, 949 74 Nitra, Slovakia; jbranisa@ukf.sk (J.B.); kjomova@ukf.sk (K.J.); radka.mala.11@gmail.com (R.M.); 2 Polymer Institute, Slovak Academy of Sciences, Dúbravská cesta 9, 845 41 Bratislava 45, Slovakia; Angela.Kleinova@savba.sk; 3 PROGRESA FINAL SK, s.r.o., Ferienčíkova 18, 811 08 Bratislava-Staré Mesto, Slovakia; volodymyr.morgunov@progresafinal.sk

**Keywords:** sheep wool, electron irradiation, point of zero charge, isoelectric point, chromium sorption, chromium complex

## Abstract

We examined the characteristics of an electron beam irradiated wool with an absorbed dose of (21–410) kGy in comparison with natural wool with respect to the determination of the isoelectric point (IEP), zero charge point (ZCP), mechanism of Cr(III) sorption from higher concentrated solutions, and the modelling of the wool-Cr(III) interaction. The data of ZPC and IEP differed between natural and irradiated samples. Increasing the dose shifted the pH of ZPC from 6.85 for natural wool to 6.20 for the highest dosed wool, while the natural wool IEP moved very little, from pH = 3.35 to 3.40 for all of the irradiated samples. The sorption experiments were performed in a pH bath set at 3.40, and the determination of the residual Cr(III) in the bath was performed by VIS spectrometry under optimized conditions. The resulting sorptivity showed a monotonically rising trend with increasing Cr(III) concentration in the bath. Lower doses, unlike higher doses, showed better sorptivity than the natural wool. FTIR data indicated the formation of complex chromite salts of carboxylates and cysteinates. Crosslinks via ligands coming from different keratin chains were predicted, preferably on the surface of the fibers, but to a degree that did not yet inhibit the diffusion of Cr(III)-cations into the fiber volume. We also present a concept of a complex octahedral structure.

## 1. Introduction

In technologies using chromium salts, adsorption can be exploited to pre-concentrate small chromium quantities for various purposes as well as in waste water management.

Considering the application of inorganic adsorption materials for chromium, Bedemo et al. [1] described the removal of Cr (III) from aqueous solution using aluminum oxide hydroxide. Park et al. [2] examined the feasibility of hexavalent chromium removal using mackinawite (FeS)-coated sand, which completely reduced Cr(VI) to Cr(III). Dong et al. [3] made poly(catechol-1,4-butanediamine)-coated Fe_3_O_4_ composite capable of successfully removing Cr(VI), some part of which could be reduced partially to Cr(III). Defective porous boron nitride was successfully tested for adsorption of Cr(III)/Cr(VI) [4], and the results clarified that the strong adsorption of both ions is chemisorption, and not dispersion or electrostatic attraction. Zhu et al. [5] prepared nanoscale zero-valent iron/nickel and tested Cr(VI) removal along with the influence of co-existing anions (CO_3_^2−^, HCO_3_^−^, SO_4_^2−^ and NO_3_^−^). The anions inhibited Cr(VI) removal, and CO_3_^2−^ had the most adverse effect.

Organic adsorbents for chromium involve synthetic or natural materials after modification. Carbonized wheat and barley straw oxidized by nitric acid showed a high adsorption towards Cr(III) [6]. Lyubchik et al. [7] prepared activated carbons for Cr(III) from co-mingled natural organic wastes. As presented by Su et al. [8], activated carbon modified with micro-sized goethite adsorbed Cr(VI) well, reducing it to Cr(III). Composite polyethylenimine-silica nanoparticles removed chromium from solution being Cr(VI), which was reduced partially to Cr(III) by amine group, whereby Cr(III) and Cr(VI) ions were adsorbed on different functional groups [9]. Mortazavian et al. [10] applied nano-scale zero-valent iron particles onto active carbon for simultaneous adsorption and reduction of Cr(VI) from aquatic solutions. Magnetic corncob biochar/polypyrrole composite applied for Cr(VI) adsorption showed combination of adsorption and reduction [11]. The presented mechanism involved (a) adsorption of Cr(VI) anions onto the surface followed by a partial anion exchange with Cl, (b) reduction of the adsorbed anions to Cr(III) by amine and hydroxyl groups, and (c) immobilization of generated Cr(III) via precipitation and chelation. Examining the biosorption potential of raw and chemically modified *Vetiveria zizanioides* grass for chromium (VI) removal, Tyagi and Khandegar [12] found that acid-modified bioadsorbents exhibited the highest efficiency. Also, Surendran and Baral [13] reported similar results for grass *Sorghastrum Nutans L. Nash*, pointing out that pH plays the most important role in Cr(VI) adsorption. Furthermore, application of several composite adsorbents for chromium is the topic of many published papers [14,15,16,17], and this could be the subject of a large review work.

As far as biosorbents of animal origin, they are practically limited to sheep wool [18,19,20]. Today, with wool being forced from the market by synthetic fibers, it is becoming an unavoidable and unwanted waste, bearing additional costs in the form of its disposal as hazardous waste. That is why some chemical modifying methods for wool have been investigated. Recent works have also presented a new radiative procedure of the modification using irradiation of sheep wool by accelerated electron beam [21,22]. Such treatment improves sorption affinity towards some metal cations [23,24,25,26]. However, much information is still unknown. The adsorption property of sheep wool is one of its many characteristics. In general, all authors dealing with adsorption processes, applied low adsorbate concentrations and obtained some standard adsorption isotherms. Since we found interesting anomalies using higher Cu(II) or Co(II) concentrations [25,26] under the sorption on the irradiated wool, this study is focused on further data gathering connected with Cr(III) sorption on natural and electron irradiated sheep wool when Cr(III) concentration was higher, too. Investigation in this field is worthwhile, because sheep wool is a renewable and cheap resource that is usable for environmental cleaning as well as valuable material recycling.

## 2. Results and Discussion

### 2.1. Impact of Absorbed Dose on Surface Properties of Wool (IEP/PZC)

Both of the parameters isoelectric point (IEP) and point of zero charge (PZC) are coessential. While the corresponding pH values are the same in metallic oxides, this is not valid for materials with other characteristics [27]. Sheep wool is also such a material, being based on keratin. Here, it is a question of the sum of the charges of the dissociated amino acid side chains on the surface of the wool fibers potentially entering electrostatic interactions (R-COO^− +^H_3_N-R).

**Figure 1:**
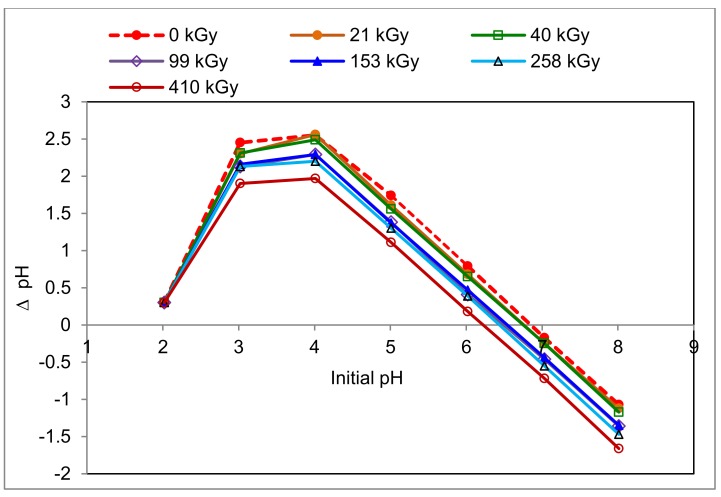
Variations of pH values in solution of 0.05 M KCl in contact with the sheep wool samples with one-year lapse from the exposure.

The varied representation of amino acids with different side chain type in keratin does not allow the determination of the IEP of sheep wool by direct calculation from pK_a_ data. Therefore, the IEP was estimated at 0.05 M KCl, similar to other biosorbents of plant origin [28], where the effect of all components is involved. In addition, we tried to find the effect of wool irradiation on the PZC and IEP position, which is identified by the electrokinetic potential ξ [29]. The data obtained are displayed in Figure 1. The wool PZCs corresponding to ΔpH = 0 were read from the measured values.

From the dependencies presented, it can be seen that the higher the dose is that has been absorbed by the wool, the lower pH will be for the PZC. The range of pH changes was from 6.85 for the non-irradiated wool to 6.2 for the 410 kGy dose. This observation is related to earlier findings that the electron irradiation of wool increases the content of acidic groups due to the cysteine acid produced by the oxidation of the cleaved sulphur bridges [21,22]. On the other hand, if the content of acidic groups (carboxylic and cysteic acids) were the only factor influencing the Cr(III) sorption of the wool, the sorption should theoretically increase with the absorbed dose. This assumption is not valid over the full range of applied concentrations (see below), which indicates that the substitution mechanism of chromium salt formation from acid residues is not the only one in the modified wool.

The pH belonging to peak on the *x*-axis corresponds to IEP. A rough reading from the graph (Figure 1) gives a uniform pH = 4.0 for IEP. In general, this value with some variations (3.3–4.5) is also reported by other authors [29,30,31]. According to Capablanca et al. [29], this figure varies with time and manner of wool scouring. When we approximated the IEP position from the intersection of the elongated side branches of the curves, slightly lower and slightly differentiated values were obtained (Table 1).

This finer reading shows a lower pH (3.35) for the non-irradiated wool than for all irradiated samples (3.40). This difference is very small, almost bordering on experimental error. However, regarding the equipment accuracy declared by the manufacturer (±0.002 pH) and relative deviation of the measured values (0.5% max.), the covering intervals of those average figures (<3.335–3.367>; <3.383–3.417>) do not yet overlap, so the difference should be accepted. In such cases, the difference should correspond to the consumed proton concentration by 4.86·10^−2^ mmol·dm^−3^. We can speculate about two possible reasons. The first is the structural disruption after the wool irradiation and related destruction of the electrostatic interactions due to conformational variations. To complete R-COO^-^ to R-COOH, the carboxyl ions bind to the H^+^ from H_2_O, decreasing the H^+^ concentration in the solution and increasing the OH^-^ concentration:

R-COO^−^ + HOH → R-COOH + OH^−^ (1)

The second reason could be due to the S-S bond being split by the electron beam, followed by oxidation of the created S-radicals with the following transformation of R-S-SO_3_^−^, R-S-SO^−^, R-S-SO_2_^−^ to R-SO_3_^−^. The latter binds to the H^+^ provided by H_2_O, giving R-SO_3_H:

R-SO_3_^−^ + HOH → R-SO_3_H + OH^−^ (2)

The hydroxyl groups resulting from both processes increase the pH value accordingly. The growth is the same for every dose, since representation of the S-oxidized products on the surface is similar. Concerning the time lapse effect [26], for other, especially shorter time periods, these values may change due to the transformation dynamics of S-oxidized species. However, in principle, the PZC and IEP will not be identical for the irradiated and non-irradiated samples.

### 2.2. Effect of pH on VIS Spektra of Cr(III)

**Figure 2:**
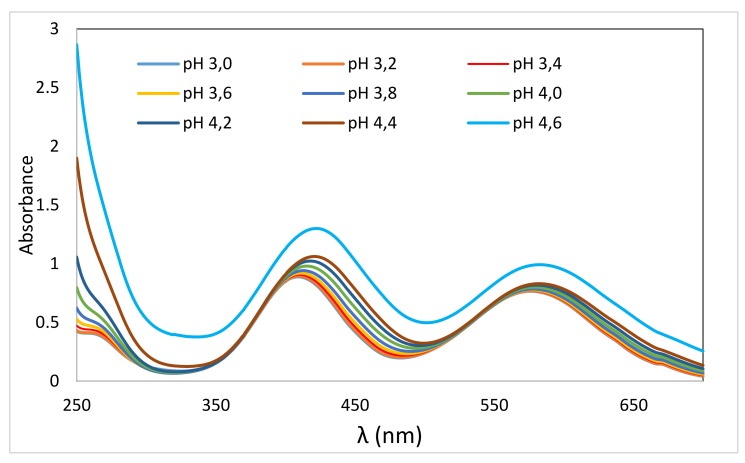
VIS spectra taken from solutions of KCr(SO_4_)_2_ (c = 50 mol.dm^−3^) with different pH values.

Changing the pH of the KCl solution with the dose (Figure 1) indicates that the resulting pH of the sorption bath will change with the absorbed dose. This may affect the VIS-spectral evaluation of residual Cr(III) content in the bath. Therefore, we verified whether and how the VIS spectrum of 50 mM KCr(SO_4_)_2_ solution changes with pH change (Figure 2) in the range of (3.0–4.6) pH units. To minimize dilution of the measured solutions, 10 M NaOH or HCl solutions were used to adjust the desired pH. After adjusting the pH to 4.8, precipitation of hydrated Cr_2_O_3_ was already observed. From Figure 2, it is evident that increasing the pH increases the absorbance and shifts λ_max_ of both absorption bands to a higher λ. The first band in the solution with pH = 3 (λ_max_ = 409 nm) corresponds to the absorbance A = 0.8877, while for pH = 4.6 (λ_max_ = 422 nm) the absorbance was measured to 1.2998. Thus, for the mentioned pH range, Δλ_max1_ = 13 nm and the increase in absorbance ΔA_1_ = 0.41. For the second band for pH = 3 (λ_max_ = 577 nm) the absorbance of 0.7648 is observed and 0.9916 for pH = 4.6 (λ_max_ = 582 nm), corresponding to Δλ_max2_ = 5 nm and ΔA_2_ = 0.227.

The increase in λ_max_ and the absorbance with increasing pH was also observed in 50 mM Cr(NO_3_)_3_.9H_2_O solution by Hamada et al. [32] when varying the pH from 2.82 to 5.05. The spectral changes are related to hydrolytic processes, to which Cr(III) has a strong tendency. The hydrolysis products are polynuclear complexes with OH-bridges. The hydroxy groups required for this are formed from coordinated water molecules by proton loss, followed by coordination of OH^−^ groups [33]. The hydrolytic process was studied by Drljaca and Spiccia [34] and Hiroishi et al. [35], who observed a supportive effect of increasing pH. Indeed, the observed spectral changes are the result of hydrolytic processes in our case as well.

Since the measurement of the Cr(III) sorption by the wool involves scanning the VIS spectra of the residual chromium in the bath, it was also necessary to take into account the pH effect demonstrated above (Figure 2) on the respective calibration curve in order to reflect the real final situation in the measured bath. The change in the initial pH of the bath based on the absorbed dose by wool was tested on 50 mM KCr(SO_4_)_2_·12H_2_O solutions after 24 h contact with each wool sample. We found that the initial pH = 3.14 for doses of (0–410) kGy varied only within a narrow range of 3.34 ± 0.02. Therefore, we set the pH of the calibration solutions to 3.34. In addition, in order to eliminate any potential interfering effects of other bath components on the spectral drift, we used an aqueous extract from the non-irradiated wool prepared in a manner consistent with the samples to prepare the calibration solutions. The calculated amounts of KCr(SO_4_)_2_·12H_2_O were dissolved in the filtered aqueous extract and the pH of the calibration solutions adjusted to 3.34 to match pH of the sorption bath. The band with λ = 583 nm was used for analysis, which appeared more stable and, in contrast to the band at λ = 415 nm, allowed a more accurate measurement of the higher concentration. Under these conditions, the calibration curve showed R^2^ = 1.0000.

### 2.3. Sorptivity

**Figure 3:**
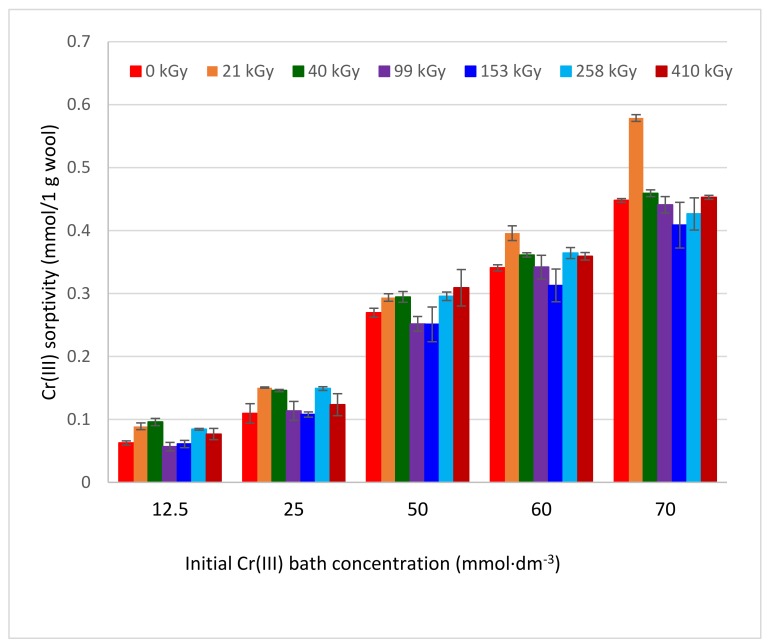
Variations of Cr(III) sorptivity onto irradiated sheep wool depending on concentration and absorbed dose.

The sorption experiments were performed with the wool of 120 days after the exposure, when the wool structure was considered sufficiently stabilized [22]. Using VIS spectral data for the band λ_max_ = 583 nm, the corresponding biosorptive dependencies were calculated (Figure 3).

All obtained dependencies were monotonically growing, without extremes, and mostly tended towards convex. They were dissimilar to the expected standard relations because the sorbent and sorbate concentrations were atypical, as was shown for Cu (II) [25] and Co(II) [26]. This may be due to the specific effect of EB modifying the fiber bulk, not just its surface. On the other hand, complexation capacity of the cations is different, too. Here, within the range of standard deviations, some of the dependencies overlap, but others are different, such as 21 kGy, especially. It can be seen that the smallest deviations are at lower concentrations. The observed differences can be attributed to sample inhomogeneity only to a small extent since the wool was taken from the identical site. While any noticeable correlation is not apparent between the irradiation dose and the Cr(III) amount sorbed within the mentioned concentration range, low concentrations of Cr(III), as well as other cations [23,24] showed a visible positive effect of the absorbed dose on the sorption. We assume the main reason for this is that the Cr(III) concentration is already high enough to create a complex salt(s) on the fiber surface affecting diffusion of the next ions into the bulk and overlapping the difference between the samples.

For simplified modelling of the interaction of Cu(II) and Co(II) with irradiated wool, the reaction with arginine was used [25,26], since arginine is one of the most abundant amino acids in keratin [36]. In both cases, the formation of soluble complex salts has been spectrally demonstrated. We used this model for Cr(III), too. However, the product of the reaction of KCr(SO_4_)_2_·12H_2_O with arginine was a relatively fast-forming greenish precipitate with a color shade similar to the wool after contact with the chromium bath.

**Figure 4:**
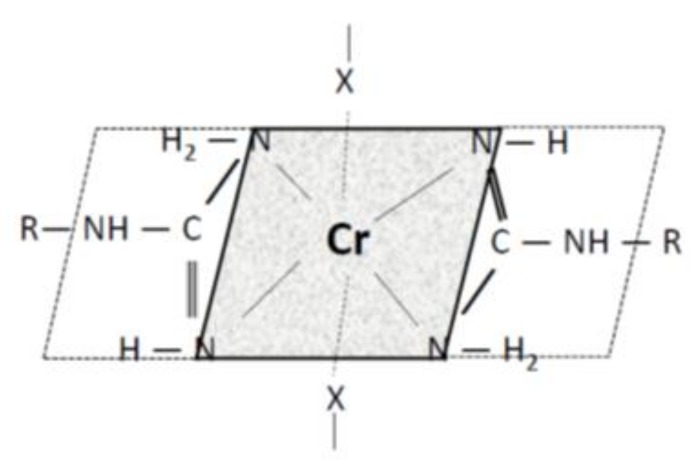
Concept of the Cr(III)-arginine complex.

Arginine (2-amino-5-guanidinopentanoic acid C_6_H_14_N_4_O_2_) is an amino acid with a basic guanidine ending and thus, in an aqueous medium, the hydrogen at the ending amino group is not acidic. The nature of the acid-base reaction of arginine is a function of the medium pH. In acidic medium at pH < IEP the carboxyl group -COOH is not dissociated and positive charges on the amino and imino groups (-NH_3_^+^) are. As a result of competition with H^+^ from the acid medium, the positive charges on the arginine ending group will repel the positively charged Cr^3+^. Above IEP at pH > IEP, the carboxyl group starts to dissociate, and as the pH increases, the dissociation deepens. Under these conditions, arginine may form a chromium salt. Since the arginine solution exhibits pH ≈ 8, the precipitate formed should correspond to chromium arginate. Moreover, since the Cr(III) atom forms complexes with all Lewis bases in practice, the precipitate represents a complex chromite salt, a chelate. All chromium complexes without exception have a coordination number of 6 and an octahedral form [37]. In the arginine-Cr(III) complex considered, regarding a planar arrangement of the guanidine moiety, it can be expected that two arginine molecules will fulfill the role of four ligands (each arginine molecule is a bidentate ligand) and two other molecules, probably water (or SO_4_^2^^−^), complete the number of ligands to six by monodentate occupations of the 2 remaining coordination sites. Such a state could correspond to the structural concept in Figure 4, where X may be a sulphate or hydroxyl anion, in the case of wool also a carboxyl or cysteinate anion:

The formation of the precipitate in reaction of KCr(SO_4_)_2_ with arginine in the aqueous solution is only a considerable simplification of the model situation in sheep wool. There are, in addition to arginine side chains, many others that can limit or modify an analogous reaction. Moreover, the presence of at least two arginine guanidine moieties at a suitable position to Cr(III) is necessary to precipitate the complex. Unlike the arginine solution, such a situation is likely to occur rarely in wool; therefore, other mechanisms also participate in the binding of Cr(III) to the fiber. If only one guanidine moiety is available as the ligand, another ligand may be provided by an amine or hydroxy group of another side chain coming from a different keratin molecule. Therefore, as with Cu (II) [25], the crosslinking of the chains must also occur in the case of Cr(III). Depending on the nature of the participating part of the keratin chain, the resulting octahedron may be the same as in the model reaction, or degenerated. The question is why at higher cation concentrations two extremes were observed on sorption isotherms for Cu (II) with comparable concentrations, unlike Cr(III), although both cations tend to form complexes. In particular, pH medium impact can be considered. The KCr(SO_4_)_2_ sorption bath has pH of ≈ 3.3, which corresponds to the IEP vicinity, while the corresponding bath pH for Cu(II) (pH ≈ 4) was higher than the IEP. In the vicinity of IEP, the dissociation of -COOH is only beginning, and deepens with increasing pH. This indicates that the situation for formation of the Cr(III) salt via substitution reaction is less favorable than that of Cu(II). Since the formation of the Cr(III) salt (carboxylate, cysteinate) is a precursor of the complex creation, fewer complex forms hindering access of further cations into the fibers will be formed in the lower pH environment. Therefore, a decrease in Cr(III) sorption is not observed in the applied concentration range. Comparing the sorption capacity of Cu(II) [25] with Cr(III) for a similar concentration interval and sorption recalculated to millimoles/g, we can see that the range of the corresponding capacity (0.11–0.236) mmol/g for Cu(II) is narrower than (0.054–0.48) mmol/g for Cr(III). This comparison supports our previous finding that the complexing ability of Cu in wool is higher than that of Cr and hampers the diffusion of ions inside the fibers causing sorption extremes.

### 2.4. FTIR Spectra Measurements

**Figure 5:**
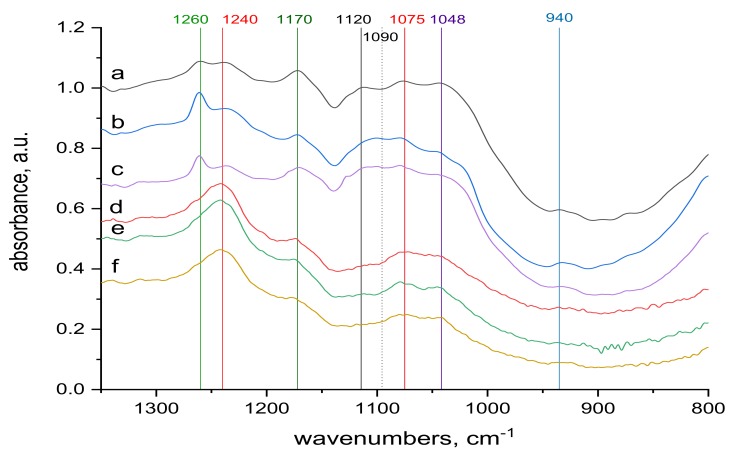
Spectra of reference materials (without KCr(SO_4_)_2_·12 H_2_O treatment), region (900−1300 cm^−1^); from up to down: (**a**) transmission spectrum of wool with 0 kGy dose; (**b**) transmission spectrum of wool with 21 kGy dose; (**c**) transmission spectrum of wool with 153 kGy dose; (**d**) ATR spectrum of wool with 0 kGy dose; (**e**) ATR spectrum of wool with 21 kGy dose; (**f**) ATR spectrum of wool with 153 kGy dose.

**Figure 6:**
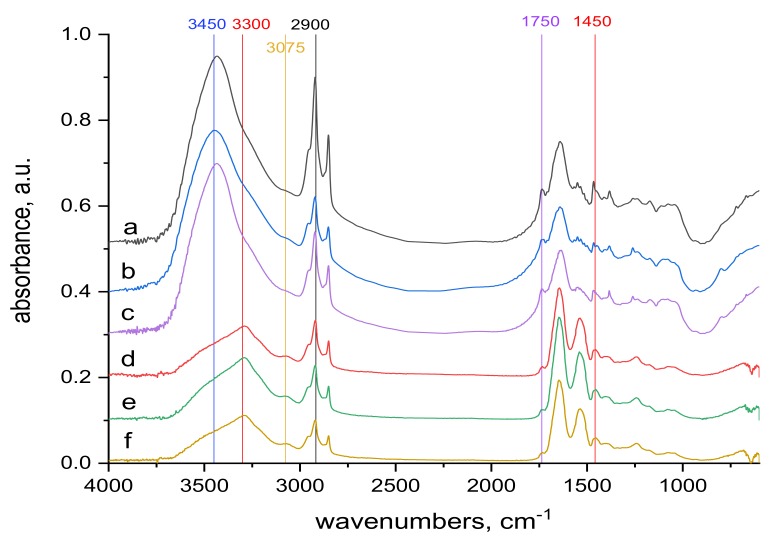
Spectra of reference materials (without KCr(SO_4_)_2_·12 H_2_O treatment), the whole mid-infrared region (400−4000) cm^−1^; from up to down: (**a**) transmission spectrum of wool with 0 kGy dose; (**b**) transmission spectrum of wool with 21 kGy dose; (**c**) transmission spectrum of wool with 153 kGy dose; (**d**) ATR spectrum of wool with 0 kGy dose; (**e**) ATR spectrum of wool with 21 kGy dose; (**f**) ATR spectrum of wool with 153 kGy dose.

**Figure 7:**
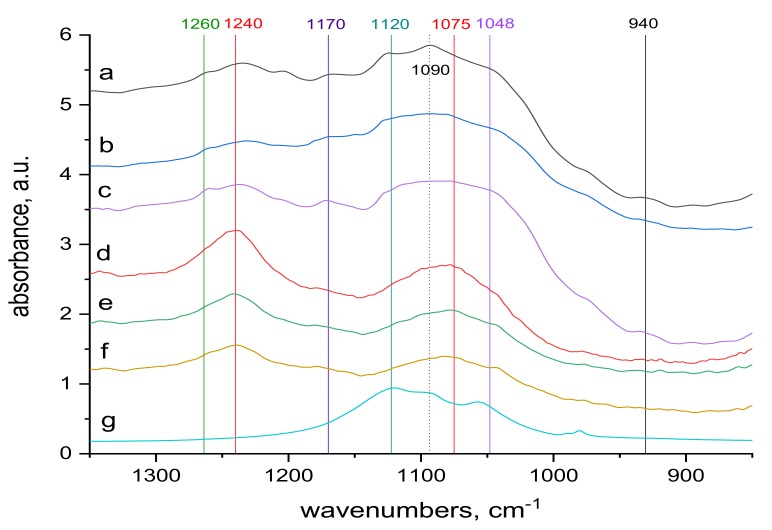
Comparison of the transmission and ATR spectra of wool with absorbed doses 0 kGy, 21 kGy and 153 kGy, respectively, after contact with 70 mM KCr (SO_4_)_2_.12 H_2_O solution, region (900–1300) cm^−1^; from top to bottom: (**a**–**c**) transmission spectra of 0 kGy, 21 kGy and 153 kGy dosed samples; (**d**–**f**) ATR spectra of 0 kGy, 21 kGy and 153 kGy dosed samples; (**g**) spectrum of KCr (SO_4_)_2_.12 H_2_O.

**Figure 8:**
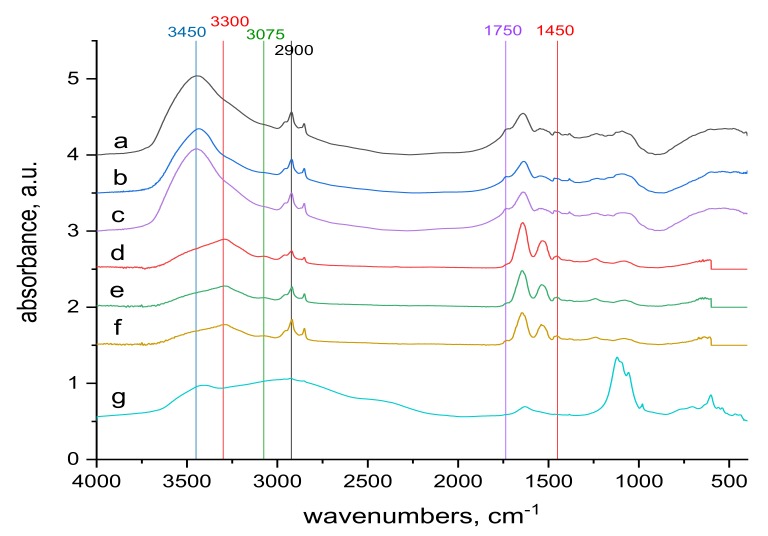
Comparison of the transmission and ATR spectra of wool with absorbed doses 0 kGy, 21 kGy and 153 kGy respectively after contact with 70 mM KCr(SO_4_)_2_·12 H_2_O solution; the whole mid-infrared region (400–4000) cm^−1^; from top to bottom: (**a**–**c**) transmission spectra of 0 kGy, 21 kGy and 153 kGy dosed samples; (**d**–**f**) ATR spectra of 0 kGy, 21 kGy and 153 kGy dosed samples, (**g**) spectrum of KCr(SO_4_)_2_·12 H_2_O.

The surface and internal wool structures are not the same. As already mentioned, the electron beam penetrates the entire wool bulk, modifying the surface as well as the internal structures. So infrared spectra can be an important contribution to qualitative understanding of the mechanism of Cr(III) sorption into both natural and irradiated wool. Comparison of the transmission spectra taken from the whole wool volume and from the surface, ATR spectra, before (Figure 5 and Figure 6) and after contact with the chromium bath (Figure 7and Figure 8) makes it possible to assess the running processes and possible differences. For better survey, in addition to the more detail spectra within the 900–1300 cm^−1^ region (Figure 5 and Figure 7), also entire the middle IR region is shown (Figure 6 and Figure 8).

**Figure 9:**
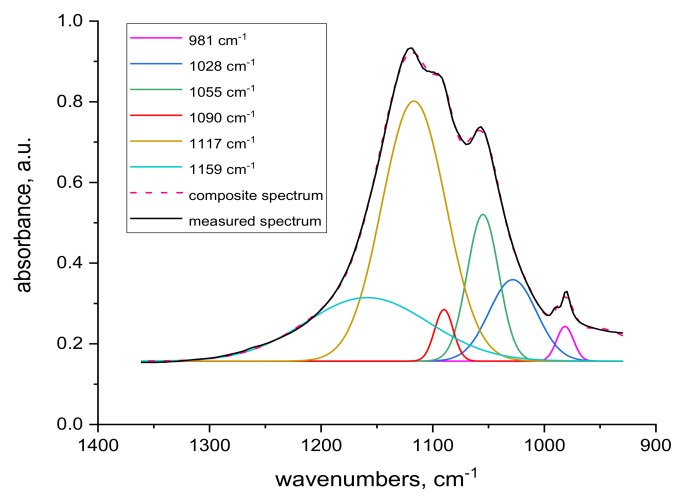
Deconvolution of transmission KCr(SO_4_)_2_ spectrum.

Spectral analysis revealed several differences. The band around 1048 cm^−1^ pertaining to cysteine acid [21,38] in both volume and sample surface is more pronounced before contact with Cr(III) (Figure 5) than after the contact (Figure 7), indicating acid consumption in reaction with the sorbate. More consistent structure of cystine monoxide bands at 1075 cm^−1^ [21] in the contacted samples (Figure 7 compared to finer structured bands of the contactless samples (Figure 5) is related to interaction of the monoxide with Cr(III). The 1090 cm^−1^ band in the non-contacted samples (Figure 5) is absent, but is noticeable in the volume of contacted samples, at most in 0 kGy sample (Figure 7a). This corresponds to sulphate anion [38,39] sorbed from the KCr(SO_4_)_2_ bath, which we verified, as documented in Figure 9, which displays the KCr(SO_4_)_2_ spectrum deconvolution.

Merging into a wider band in samples of 21 and 153 kGy (Figure 7b,c) indicates a wider involvement of this anion in the interaction due to the structural changes after the irradiation. The band around 1120 cm^−1^ belonging to cystine dioxide [21] is indicated in the non-contacted sample in the volume of the non-irradiated sample and disappears with increasing dose (Figure 5a–c); it is hardly noticeable on the surface (Figure 5d–f). This is because the absorbed energy of electron beam already causes the formation of cysteine acid as the final oxidation product at the expense of cystine dioxide. In the contacted samples, the situation is the same, although the absorption maximum of KCr(SO_4_)_2_·12 H_2_O in this region may distort the situation. A well readable band at 1170 cm^−1^ in the spectra of the non-contacted sample (Figure 5a–f) indicates cysteic acid [38,39] and cystine oxides both in volume and on the sample surface. Corresponding bands of the contacted samples are less pronounced in volume, at most in the 153 kGy sample (Figure 7a–c), while only a tip-off on the band is visible on the surface (Figure 7d–f). This demonstrates the consumption of these species as a result of their interaction with Cr(III), especially on the fiber surface. The bands at 1240 and 1260 cm^−1^ characterize the conformational forms of the secondary wool structure [21]. On the surface of the non-contacted wool (Figure 5d–f) there are distinct bands at 1240 cm^−1^ belonging to Amide III (mixed NH vibrations) in β-sheet and disordered structure [21] and are observable as adjacent bands of α-helical structure bands at 1260 cm^−1^ (Figure 5a–c). It can be observed that with respect to the volume of the non-contacted samples, the ratio of the bands at 1260 to 1240 cm^−1^ increases with increasing dose (Figure 5a–c), indicating the transformation of the β-sheet and disordered structure into an α-structure. In the contacted wool volume, these α-conformation bands (1260 cm^−1^) are almost unidentifiable (Figure 7a–c), and there is no sign of α-helical structure on the surface (Figure 7d–f). Although in volume of the contacted wool, the presence of a rest of the α-structure (1260 cm^−1^) is only indicated, it is apparent that the ratio of the bands at 1260 to 1240 cm^−1^ increases with dose (Figure 7a–c) even if the initial values are significantly different from the non-contacted sample. Thus, the morphology of this doublet varies with dose, and Cr contact, among other factors, affects the overall sorption result.

The 1450 and 1750 cm^−1^ bands belonging to the carboxyl -COOH [38] are identifiable in the volume and on the surface of both sample types. The reduction of the band in the contacted samples (Figure 8) compared to the starting samples (Figure 6) corresponds to the consumption of a portion of the carboxyl in reaction with Cr(III). Another band where changes can be identified is the band at 3300 cm^−1^ (Figure 6 and Figure 8). It belongs to bonded -OH and bonded -NH or -NH_2_ groups [38]. It is observable as a wider band only on the surface of both sample types (Figure 6d–f and Figure 8d–f). When compared with alkyl bands around 2900 cm^−1^ (considered constant) in the non-contacted samples (Figure 6d–f), for 0 kGy sample the absorption ratio at 3300 cm^−1^ to alkyl bands is almost equal and with increasing dose this ratio slightly rises in favor of 3300 cm^−1^. In the contacted samples (Figure 8d–f) this development is different; in the 0 kGy sample, the 3300 cm^−1^ band is higher than the alkyl band, in 21 kGy, ratio of the 3300 cm^−1^ to the alkyl band becomes equal, and at 153 kGy, the alkyl band is higher than the 3300 cm^−1^ band. The different tendencies observed are attributed to the gradual involvement of -OH, -NH or -NH_2_ groups in interaction with Cr(III) to form complex (es). Small bands at 3075 cm^−1^, measured only on the surface of both non-contacted (Figure 6d–f) and contacted (Figure 8d–f) samples, may belong to free secondary amines or bonded -OH in carboxylic acid [38]. They correspond with the large band at 3450 cm^−1^ belonging to bonded -OH and free -NH or -NH_2_ groups [38] observed only in the volume of these samples (Figure 6a–c and Figure 8a–c). This indicates that the interaction of Cr(III) with the wool takes place mainly on the surface.

## 3. Materials and Methods

### 3.1. Chemicals

Chromium potassium sulphate dodecahydrate KCr(SO_4_)_2_·12H_2_O p.a., supplied by Gavax, Ltd., (Vranov n/Topľou, Slovakia) was used as Cr(III)-sorbate. For sorption experiments, solutions of Cr(III) in deionized water at concentrations of (12.5-25-50-60-70) mmol·dm^−3^ were applied.

Natrium hydroxide NaOH, hydrochloric acid HCl and potassium chloride KCl, all analytical grade, were supplied by Slavus Ltd. (Bratislava, Slovakia) and used to adjust the pH of the solutions when necessary.

l-Arginine 98% was supplied by company Alfa Aesar, Karlsruhe, Germany.

### 3.2. Preparation of Adsorbent

Sheep wool of Suffolk-Merino crossbreed bred in West Slovakia comes from spring cut. The wool was taken from the saddle part and the fiber thickness was in the range of 27–33 μm. The wool was firstly scoured in tepid water and then thrice in an ultrasonic bath of 40 °C during 10 min, rinsed with deionized water, and freely dried for 3 days. Irradiation of the samples stored in cardboard boxes was performed in UELR-5-1S linear electron accelerator (FGUP NIIEFA, Petersburg, Russia) with installed energy of 5 MeV and operated by Progresa Final SK company (Bratislava, Slovakia ). The samples with absorbed doses of (0-21-40-99-153-258-410) kGy were stored under common conditions and room temperature.

### 3.3. Procedure of PZC and IEP Estimation

The point of zero charge (PZC) indicates the pH value at which an adsorbent submerged in an electrolyte exhibits zero net electrical charge on the surface [40]. Below the PZC, the adsorbent surface is positively charged and attracts anions. Conversely, above PZC the surface is negatively charged and attracts cations/repels anions. Isoelectric point (IEP) is known as the pH at which both positive and negative charges on the different functional groups balance each other out [41]. While for simple amino acids IEP can be estimated by calculation from pK_a_ data, for proteins, the IEP is understood to be the point at which the hydrogen ion concentration of a solution in which the ionization of the acid groups of the amphoteric substances, ampholytes, is equal to the ionization of the basic groups [30]. Here, the IEP estimation can be performed by measuring the change in pH of the wool extract in a neutral salt solution with set initial pH value following [28].

A set of samples with a one-year lapse from irradiation was selected for the experiment. The samples were contacted with 0.05 M KCl solutions whose pH was adjusted from 2 to 8. After 24 h contact, the resulting pH of the potassium chloride bathes was measured to determine its change from the set value.

The value of pH was measured using Orion2 Star pH-meter (Thermo Scientific, Waltham, MA, USA) equipped with Sen Tix 42 plastic electrode with temperature sensor. The accuracy of the equipment indicated by the manufacturer was ±0.002 pH. Applying double measuring the relative error did not exceed 0.5%.

### 3.4. Spectral Measurements

Visible spectrometry (Specord 50 Plus, Analytikjena, Germany) with a 1 cm cell was used to determine the Cr(III) residual content in the bath. The comparative sample was always the aqueous extract from the wool with dose corresponding to the measured sample, obtained after 24 h contact of the sample with deionized water under the same conditions.

Infrared spectrometry was applied to analyze the wool samples before and after contact with Cr(III). Fourier transform infrared spectroscopy-attenuated total reflectance (FTIR-ATR) measurements were performed with a NICOLET 8700TM FTIR™ spectrometer (Thermo Scientific, Waltham, MA, USA) using a single bounce ATR accessory equipped with a Ge crystal. For transmission measurements the fibers were immersed directly into liquid nitrogen for 5–10 min and then ground in the ball mill. The ground powder in an amount of 0.9–1.9 mg was molded into KBr pellets. The corresponding spectra were taken throughout the whole middle infrared region (400–4000 cm^−1^) and normalized by converting to unit mass. In case of KCr(SO_4_)_2_·12H_2_O, the quantity was 1.7 mg. For each measurement, the spectral resolution was 4 cm^−1^ and 64 scans were performed. The acquired spectra were analyzed using the OMNIC™ v.8.1 spectroscopic software (Thermo Electron Scientific Instruments LLC, Madison, WI, USA).

### 3.5. Batch Sorption Experiments

The sorption experiments were conducted with Cr(III) solutions applying concentrations in the range of (12.5–70) mmol·dm^−3^. After being cut to 3–5 mm, 0.2 g of wool fibers was placed into a glass cup with a cap and the testing solution of 20 cm^3^ in volume was added. The content of the glass cup was first shaken for 6 h at room temperature on a laboratory horizontal shaker (Witeg SHR-2D, Labortechnik GmbH, Wertheim, Germany) and then kept in static mode for next 18 h. Then the remaining solution was filtered through KA5 filter paper and used for determination of residual Cr(III). Every sorption procedure was carried out in triplicate.

The parameter q as a measure of wool (bio)sorptivity was calculated using the following equation:

q = (x_1_−x_2_)/m (3)

where q is the sorptivity defined as the amount of sorbate in mmol per 1 g of the sorbent for individual wool samples when particular testing solution is applied in specified concentration, x_1_ is the amount of the sorbate added in the initial solution (mmol), x_2_ is residual amount of the sorbate in the solution after its contact with the wool sample (mmol), m is the mass of the wool sample taken for analysis (g).

## 4. Conclusions

Sheep wool, both natural and electron beam irradiated with absorbed doses of (0–410) kGy, was subjected to PZC and IEP examination using pH change in 0.05 M KCl solutions with adjusted pH after the contact with the wool samples. The increasing dose shifted PZC to lower values with an overall pH drop in the range of 0.65 pH units, while the IEP for the irradiated wool increased by 0.05 pH units, equally for all dosed samples compared to pH of 3.35 for the non-irradiated wool. In the sorption experiments with KCr(SO_4_)_2_.12H_2_O, the influence of hydrolytic processes of the sorbate on the change of the VIS spectrum used for the evaluation of the Cr(III) sorption by the wool was taken into account. This was eliminated by using a pH adjusted aqueous wool extract corresponding to the resulting pH of the sorption bath and used as a solvent for the preparation of the calibration solutions. The calculated sorptivity for Cr(III) in the range of (12.5–70) mM concentration showed a monotonically rising trend without extremes despite the expected analogy with the sorption of Cu(II) or Co(II) showing extremes. Compared to the non-irradiated wool, lower doses (21–40 kGy) induced better sorption than those higher doses. The model reaction of Cr(III) with arginine resulted in an insoluble chelate, which was used to draft a concept of the octahedronic Cr-complex model. Infrared spectral data from the surface and volume of the wool fibers confirmed Cr(III) chemisorption involving the formation of complex chromite salts based on carboxylates and cysteinates. The morphology of bands of secondary structure (α, β, amorphous) changed with the position in the fiber (surface/volume) and dose, as well as due to the interaction with Cr(III). The amino- and hydroxyl-type ligands provided by various chains are precondition for cross-linking formation. The interaction of Cr(III) with the wool was indicated preferentially on the fiber surface.

## Figures and Tables

**Table 1:** Position of isoelectric point (IEP) for sheep wool with one-year lapse from electron beam irradiation. The IEP is estimated by approximation from Figure 1.

**Dose absorbed by wool (kGy)**	0	21	40	99	153	258	410
**IEP as pH_max_ estimated from Figure 1**	3.35	3.40	3.40	3.40	3.40	3.40	3.40
